# Poisson-Like Spiking in Circuits with Probabilistic Synapses

**DOI:** 10.1371/journal.pcbi.1003522

**Published:** 2014-07-17

**Authors:** Rubén Moreno-Bote

**Affiliations:** 1 Research Unit, Parc Sanitari Sant Joan de Déu and Universitat de Barcelona, Esplugues de Llobregat, Barcelona, Spain; 2 Centro de Investigación Biomédica en Red de Salud Mental (CIBERSAM), Esplugues de Llobregat, Barcelona, Spain; NTNU, Norway

## Abstract

Neuronal activity in cortex is variable both spontaneously and during stimulation, and it has the remarkable property that it is Poisson-like over broad ranges of firing rates covering from virtually zero to hundreds of spikes per second. The mechanisms underlying cortical-like spiking variability over such a broad continuum of rates are currently unknown. We show that neuronal networks endowed with probabilistic synaptic transmission, a well-documented source of variability in cortex, robustly generate Poisson-like variability over several orders of magnitude in their firing rate without fine-tuning of the network parameters. Other sources of variability, such as random synaptic delays or spike generation jittering, do not lead to Poisson-like variability at high rates because they cannot be sufficiently amplified by recurrent neuronal networks. We also show that probabilistic synapses predict Fano factor constancy of synaptic conductances. Our results suggest that synaptic noise is a robust and sufficient mechanism for the type of variability found in cortex.

## Author Summary

Neurons in cortex fire irregularly and in an irreproducible way under repeated presentations of an identical stimulus. Where is this spiking variability coming from? One unexplored possibility is that cortical variability originates from the amplification of a particular type of noise that is present throughout cortex: synaptic failures. In this paper we found that probabilistic synapses are sufficient to lead to cortical-like firing for several orders of magnitude in firing rate. Moreover, the resulting variability displays the property that variance of the spike counts is proportional to the mean for every cell in the network, the so-called Poisson-like firing, a well-known property of sensory cortical firing responses. We finally argue that far from being harmful, probabilistic synapses allow networks to sample neuronal states and sustain probabilistic population codes. Therefore, synaptic noise is not only a robust mechanism for the type of variability found in cortex, but it also provides cortical circuits with computational properties to perform probabilistic inference under noisy and ambiguous stimulation.

## Introduction

Cortical neurons respond to repeated presentations of the same stimulus in a remarkably idiosyncratic way, and no identical responses are observed twice [1], [2], [3], [4]. Although the spike count responses are on average reproducible (*ibid*.), they display high variability. It is well established that in evoked conditions the variance of the spike count over time windows of a few hundreds of milliseconds is closely proportional to the mean spike count, which in turn implies that the Fano factor –variance to mean ratio– is approximately constant as a function of firing rate [2], [3], [5], [6], [7]. Approximate Fano factor constancy is not only found for a small range of evoked firing rates, but rather it holds for the whole observable dynamic firing range of cortical neurons, which covers from a few to hundreds of spikes per second [2], [5], [7], [8]. In addition, Fano factor constancy is not just a property of the distribution of a neuronal population, but every single neuron in the population displays Fano factor constancy over its whole dynamical range [3], [7]. This single-cell, whole dynamical range property is referred here to as Poisson-like firing, in analogy to the Poisson process, whose Fano factor is rate-independent.

Theoretical neuronal network models often invoke a balance between excitatory and inhibitory inputs to describe the high spiking variability observed in cortex, a mechanism that leads to complex or chaotic firing behavior that is Poisson-like at low firing rates [3], [9], [10], [11]. These networks can also be adapted to display bistable dynamics to model working memory tasks, and it has been shown that they can generate Poisson-like firing during persistent activity even at moderately high firing rates [12], [13], [14], [15], [16], [17]. Relatively less attention, however, has been paid to study the origin of Poisson-like variability over a full continuum (non-discrete) of firing rates ranging from a few to hundreds of spikes per second [18], as observed experimentally in sensory areas [3], [9], [10], [11]. As we show below, although balanced excitatory and inhibitory networks are well suited to generate Poisson-like firing at low rates, balanced networks fire with low variability as their firing rate increases continuously unless connectivity parameters are fine-tuned [12] or inputs to the network are themselves Poisson-like [18]. Introducing Poisson-like inputs to obtain Poisson-like outputs is a valid solution to the problem of how variability is generated in cortex. However, this solution might seem unsatisfactory because it does not address the problem of how and where Poisson-like inputs are originated in the first place. Moreover, the notion of Poisson-like inputs to sensory cortical areas fails to find strong experimental support, because LGN spike train inputs to V1 display Fano factors decreasing by two-fold or more as a function of firing rate [19], [20]. In summary, although there is a solid understanding of how Poisson-like variability arises from the chaotic balanced dynamics of neuronal networks at low rates [3], [9], [10], [11], or in discrete high rate persistent states [12], [13], [14], [15], the mechanisms underlying single-cell Poisson-like variability over a broad continuum of rates have not yet been elucidated.

Other sources of noise in neuronal networks that have so far been largely neglected might be responsible for cortical spiking variability, in particular at high firing regimes. A well-documented source of variability in the central nervous system is synaptic transmission failures [21], [22], [23]. Synaptic vesicle recovery and release has complex time history dependences [24], [25], [26], [27], but at the finest level synaptic transmission is fundamentally probabilistic [21], [22]. In this paper, we show that amplification of synaptic noise generated by realistically small postsynaptic potentials through recurrent connections is sufficient to generate Poisson-like spiking over several orders of magnitude in the firing rate. Other variability-inducing mechanisms, such as random synaptic delays or intrinsic spike generation jittering [28], [29], constitute negligible sources of spiking variability at high rates.

## Results

### A single neuron case

**Figure 1:**
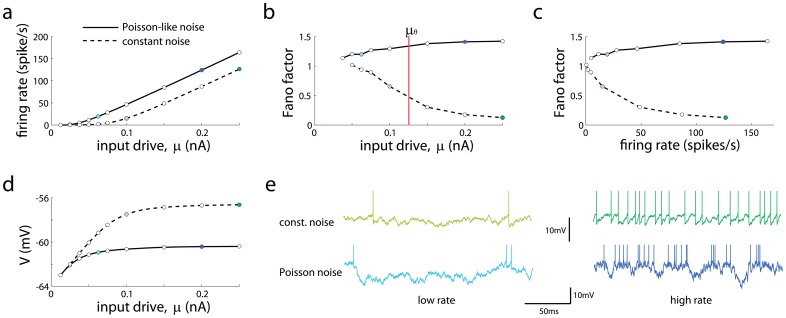
Poisson-like output firing requires Poisson-like inputs in a single neuron. (**a**) Firing rate as a function of mean input drive with constant noise (dashed lines) and Poisson-like input noise 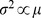 (solid lines). (**b,c**) Fano factor as a function of mean input drive (b) and firing rate (c). Vertical red line indicates threshold current 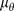, defined as the minimal current to elicit firing in absence of input noise. (**d**) Mean membrane potential as a function of mean input drive. (**e**) Membrane potential traces corresponding to color dots in the previous panels: low (light green) and high (dark green) firing rate with constant input noise, and low (light blue) and high (dark blue) firing rate with Poisson-like input noise. Low and high firing rates conditions were chosen such that firing rates were comparable for the two input noise types.

To understand under what conditions Poisson-like firing can be generated, we simulated a single leaky integrate-and-fire neuron with various types of input white noise. We first considered the case where the mean input variance is constant as the mean input rises (Fig. 1, dashed lines). In correspondence to the responses of sensory cells to stimuli with increasing intensity (e.g. contrast in V1 [5], [30]), boosting the mean input drive of the neuron increases its firing rate and mean membrane potential (Fig. 1a,d). Although the Fano factor is close to one at low input drive (Fig. 1b, dashed line), when the input drive is above the threshold 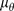 (vertical line) the Fano factor drops to very small values. This in turn implies that the Fano factor decreases to low values at high rates (>50 Hz) (Fig. 1c, dashed line). At low rates the mean current is below the threshold current (sub-threshold regime) and spiking is induced by membrane potential fluctuations (Fig. 1e, light green trace), while at high rates the mean current is above the threshold current (supra-threshold regime) and spiking is mainly induced by voltage threshold crossings around the mean membrane potential trajectory (dark green trace), leading to a very regular spike train with low Fano factor.

As constant input noise was not able to keep high the Fano factors at high rates, we considered next the scenario where the input variance grows in proportion to the mean input current (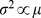). This manipulation corresponds to the case where inputs are Poisson-like because a rate-independent Fano factor in the input spike trains implies proportionality between input variance and mean [31]. In this scenario, the output Fano factor remains approximately constant even at very high rates (>100 Hz) (Fig. 1c, solid line). Like the constant input noise case, at low rates spikes are induced by small membrane potential excursions around the mean membrane potential trajectory that sporadically reach spiking threshold (Fig. 1e, light blue trace). However, unlike the constant input noise case, at high rates the membrane potential undergoes large fluctuations that cause bursts of spikes followed by silence periods (dark blue trace), leading to high spiking variability even at elevated firing.

The two scenarios described above are *a priori* possible in recurrent networks. If the Fano factor of the afferent spike trains to a neuron in the network decays with firing rate as 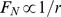 (as in dashed line of Fig. 1c), the input noise becomes approximately constant because 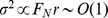 [31]. In this scenario we find that the Fano factor of the output spike train is 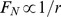, and therefore the Fano factor displays the same firing rate scaling as that in the inputs. If, in contrast, the Fano factor of the afferent spike trains is constant with firing rate, 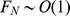, then the input noise becomes Poisson-like because 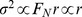. In this scenario the Fano factor of the output spike train is approximately independent of firing rate, 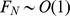 (solid line in Fig. 1c), like the Fano factor in the input, and it again displays the same firing rate scaling as that in the inputs. Therefore the two scenarios are potentially self-consistent in the sense that the same type of variability that is introduced in the inputs is recovered in the outputs.

### Breakdown of Fano factor constancy

**Figure 2:**
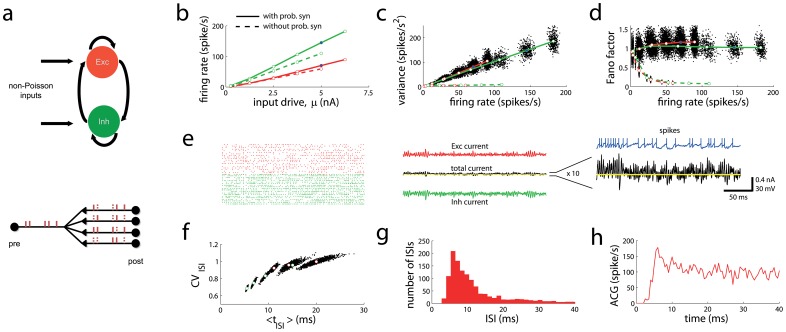
Approximate Fano factor constancy with probabilistic synapses. (**a**) Scheme of a balanced recurrent network with excitatory and inhibitory neurons driven by non-Poisson-like inputs. *Bottom*: the network is embedded with probabilistic synaptic transmission. The scheme shows how a presynaptic spike train generates stochastic currents on several postsynaptic neurons. (**b**) Mean firing rate for excitatory (red) and inhibitory (green) populations for a network with probabilistic synapses and noiseless inputs (solid lines) and for a network without probabilistic noise and constant input noise (dashed lines) as a function of the mean input drive. (**c–d**) Spike count variance and Fano factor as a function of firing rate. Open circles correspond to mean values, and black dots correspond to individual neurons. Line and color codes are as in panel b. (**e**) Raster plots of 20 randomly selected excitatory and inhibitory neurons for the high firing rate network corresponding to the point marked in blue in panels b–d. *Center*: sample traces of excitatory and inhibitory current leading to the net input current (black), magnified on the right. Yellow line corresponds to zero net current, and blue trace shows the membrane potential of a randomly selected excitatory neuron. (**f**) Coefficient of variation of the ISIs, 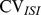
_,_as a function of the mean ISI. (**g**) Distribution of ISIs for the selected neuron. (**h**) Auto-correlogram (ACG) of the spike train for that neuron.

We sought to determine what type of neuronal variability is self-consistent and stable in recurrent networks, that is, whether the Poisson-like input noise scenario or the constant input noise scenario described above is stable in a recurrent network. We simulated a balanced recurrent spiking network [9], [10], [32] that generated strong excitatory and inhibitory currents. We also stimulated the network with external inputs. The external inputs were designed to be non-Poisson-like because the central question is whether Poisson-like variability can be self-generated by neuronal networks when the external inputs are not in the same Poisson-like family (Fig. 2a, top). The results shown below correspond to networks with non-Poisson-like inputs modeled with constant variance to enforce the experimental constraint that the input Fano factor decreases with firing rate [19], [20].

**Figure 3:**
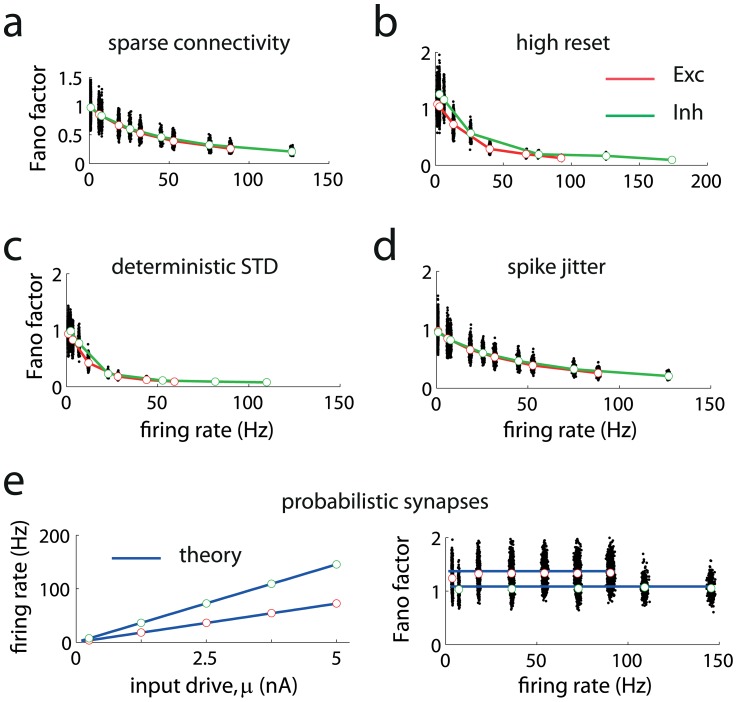
Sparse connectivity, high reset voltage or deterministic STD does not necessarily produce Poisson-like firing. (**a**) Sparse and randomly connected networks display low spiking variability at high rates. (**b**) Raising the reset membrane potential of the neurons increases the Fano factor at low rates but does not generate Poisson-like firing for a broad range of firing rates. (**c**) Networks with deterministic STD fail to generate Poisson-like variability at high firing rates. (**d**) Networks with random spike jittering display low firing variability at high rates. (**e**) Exact analytical predictions for networks with probabilistic synapses without STD (blue lines) for the firing rate (left) and Fano factor of the spike counts (right). Red and green points correspond to simulations results for excitatory and inhibitory neurons, respectively. Blue solid lines correspond to theoretical predictions.

As the input drive increases, the mean firing rates (Fig. 2b, dashed lines) for the excitatory (red) and inhibitory (green) populations increase accordingly. At low firing rates the Fano factor is high (Fig. 2c,d, dashed lines), a standard property in neuronal networks in the balanced regime [9], [10], [32]. However, the Fano factor drops monotonically to very low values as the mean population rate increases (above 50 spikes per second). As long as the network holds a single stable state (see Methods), the breakdown of approximate Fano factor constancy at high rates occurs regardless of the connectivity matrix of the network, including sparsely, densely and fully connected networks (see below); it also occurs regardless the overall connection strength, that is, the type of synaptic strength scaling used as the network becomes large, and regardless of the intensity of the constant noise. If the network is multi-stable, transitions between different states can exist, but conditioned on each state, the Fano factor is very low. These results are shown analytically for an even broader family of spiking neuronal networks in the Methods (see eqs. (11) and (22)). In particular, for sparse and randomly connected balanced networks the dynamics displays elevated Fano factors at low rates [9], [10], [33], but at high rates (>50 spikes per second) Fano factors fall off (Fig. 3a). This is because the neurons in the network enter in the supra-threshold regime, in which firing is mainly induced by mean membrane potential threshold crossings; as a result variability becomes progressively lower as firing rates increases (Fig. 3a). The same results hold when the network is randomly but densely connected and when the network is fully connected (see Fig. 2d, dashed lines). In addition, if the value of the reset membrane potential is raised, the variability increases at low rates, but the Fano factor does not remain steady at high rates (Fig. 3b). In conclusion, the previous analysis manifests that under a broad range of situations and network designs, the constant input noise scenario (corresponding to Fano factor decreasing monotonically with firing rate) becomes the only stable scenario in recurrent networks.

### Poisson-like variability in networks with probabilistic synapses

The previous results suggest that, at least at high rates, additional sources of variability are required to account for Poisson-like spiking. A well-known source of noise in cortex is probabilistic synaptic transmission. Neurotransmitter release at a synapse upon arrival of an action potential is fundamentally stochastic [21], [22] and thus it will result in spiking variability (Fig. 2a, bottom). However, it is not obvious that this source of noise can account for most of the spiking variability observed in cortex. The average number of contacts that a cortical neuron makes on postsynaptic targets is 2–6 [22] and synaptic release is independent across contacts. Therefore it could occur that synaptic noise is mostly averaged out, leaving very little room for its contribution to spiking variability. Whether strong amplification of synaptic noise can be achieved with realistic neurophysiological parameters and whether probabilistic synapses can give rise to Poisson-like variability is unknown.

We studied a balanced recurrent network with probabilistic synapses where the probability that an action potential generated a post-synaptic current underwent stochastic short-term-depression (STD) (see Methods). The network can generate high spiking variability for its full dynamical range when the connections are sufficiently strong even when the external input to the network is noiseless (Fig. 2c, solid lines; see also Fig. 2e, left panel). In the network, a presynaptic spike caused postsynaptic potentials between 0.2 and 1 mV on average, within the neurophysiological range [34], [35]. Therefore weak, independent noise across synaptic contacts can be amplified by strong synapses to generate high fluctuations at the spiking level. The network was not only able to generate high variability, but the Fano factor was approximately constant for at least two orders of magnitude range in firing rate (Fig. 2d, solid lines). Importantly, the Fano factor was not only constant on average over the population (white dots), but also individually for each neuron (black dots) as a function of firing rate.

**Figure 4:**
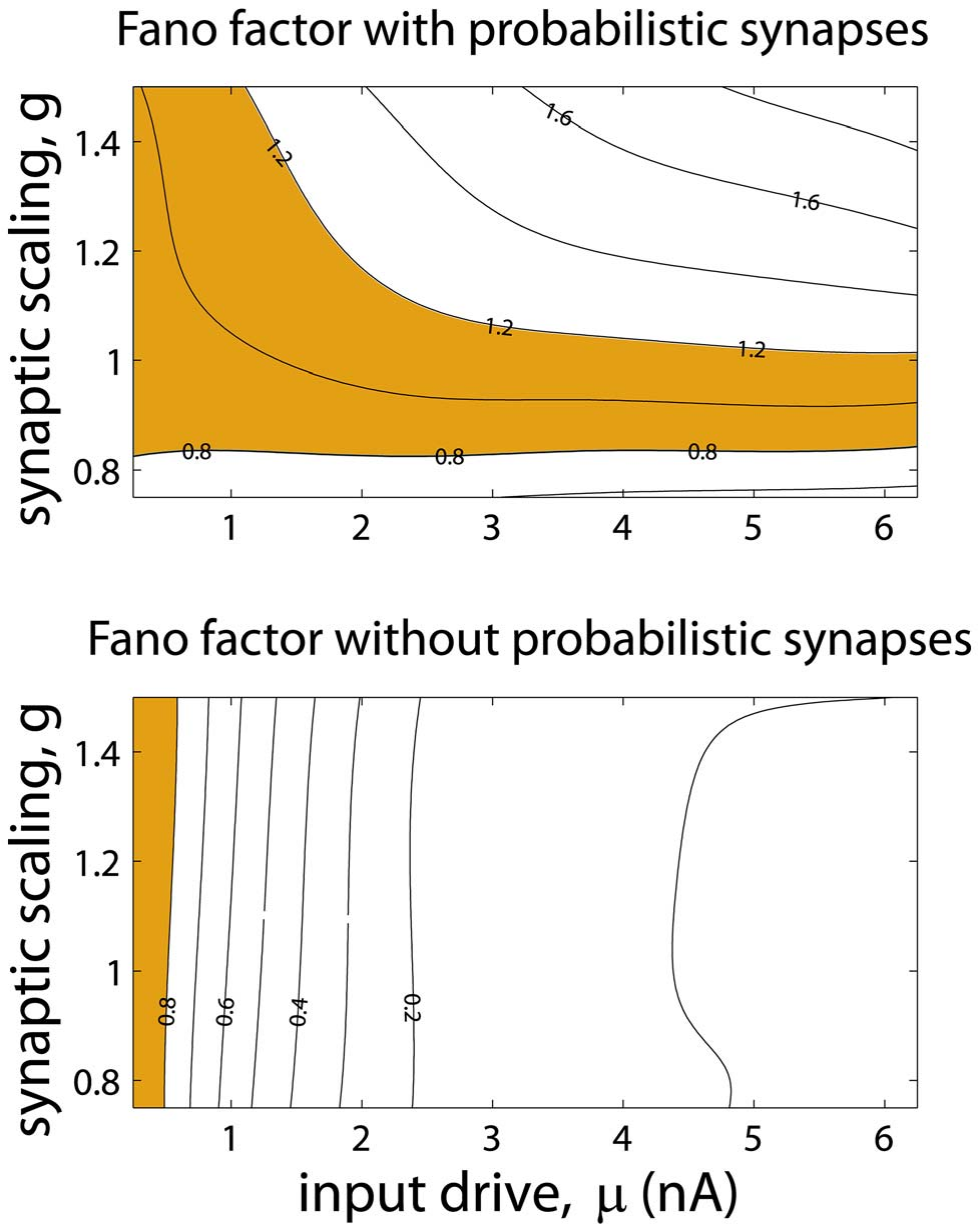
Poisson-like variability from probabilistic synapses does not require fine-tuning of the parameters. The plots display the iso-Fano factor lines on the synaptic scaling factor *g* vs. input drive plane for a network with (top) and without (bottom) probabilistic synapses. The region for which the Fano factor is high and sustained (shaded area) is broad for a network with probabilistic synapses, but this region vanishes at moderately high rates for a network without probabilistic synapses. Network parameters are as in Fig. 2. The shaded areas are defined as the areas of the planes with Fano factors lying between 0.8 and 1.2.

The neurons' Fano factors increase with the strength of the recurrent connections, but in all cases they remain constant at high rates (shown analytically for general neuronal networks in the Methods). The Fano factor was high and sustained for a broad region of scaling factors of the synaptic strength and input drives (Fig. 4, top panel), but this region vanished at moderately high input drives when the network lacked probabilistic synapses (lower panel). These results hold when synapses display STD dynamics as long as synaptic transmission does not saturate for a very broad range of firing rates [23], [36] (see Methods). When STD is modeled without stochastic release [26], [27] the Fano factor decreases monotonically to very low values at high rates (Fig. 3c). Although high reset and STD are required in some models of delayed persistent activity to generate high variable binary attractor states [13], these mechanisms do not guaranty high variability for a broad continuum of rates, as it has been shown above (Fig. 3b,c). Finally, Poisson-like variability also holds for a stochastic model of synaptic transmission without STD (Fig. 3d; see eqs. (11) and (22) in Methods). In summary, the probabilistic nature of synaptic transmission is sufficient to robustly generate Poisson-like firing at high rates.

At elevated rates (blue dots in Fig. 2b–d), as it is the case at low rates, the network generates strong excitatory and inhibitory currents (Fig. 2e, red and green traces in the middle panel, respectively) that approximately cancel, leading to a balanced net input current (black trace) that wanders around zero (yellow line). However, the net input current is on average above zero and close to the threshold current (mean 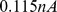; threshold current 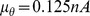). For the rightmost point in the solid lines of Fig. 2b–d, the mean current is supra-threshold. As it has been shown for single neurons (see Fig. 1), supra-threshold currents or currents around threshold generate low Fano factors unless the input noise is Poisson-like. Here, at elevated rates the net current is close or within the supra-threshold regime, and therefore the network must have generated spontaneously a current that is in the Poisson-like family (see next section).

Further support for our theory arises from the puzzling dependences of other statistical measurements of variability with firing rate. It is well established that the coefficient of variation (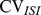) of the inter-spike-intervals (ISIs) of cortical neurons (s.d. to mean ratio) decreases at high firing rates [3], [4]. The rate dependence of 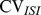 seems to be at odds with the Fano factor constancy at the same high firing rates. In fact, if spike trains were renewal processes, one would expect that the Fano factor were equal to 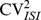. Although renewal point processes with absolute spiking refractory periods could explain the drop of 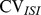 at very high rates [4], they cannot explain why the Fano factor does not decay at high rates in the same way. In our recurrent networks with probabilistic synapses, the 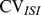 increases with the mean ISI (Fig. 2f), implying that it decreases as a function of the firing rate. This is so even when the Fano factor remains approximately constant for the whole range of firing rates, particularly at high rates (see Fig. 2d). The reason for this behavior is that the network dynamics generates temporal correlations that make the spike trains non-renewal, with experimentally consistent ISI distributions and auto-correlations functions (Fig. 2g,h). Therefore, a network effect that cannot be understood at the single neuron level gives rise simultaneously to approximate Fano factor constancy and the drop of 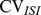 at high rates (equivalently, at short mean ISIs). Finally, although no fit of the experimental data was performed, the dependence of the 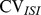 as a function of the mean ISI followed well the values and the mean ISI dependence previously reported [4], with values close to one above mean ISIs of 30 ms, and a reduction of variability up to a value of around 0.6 at mean ISIs shorter than 20 ms.

### Mechanism for Poisson-like variability

**Figure 5:**
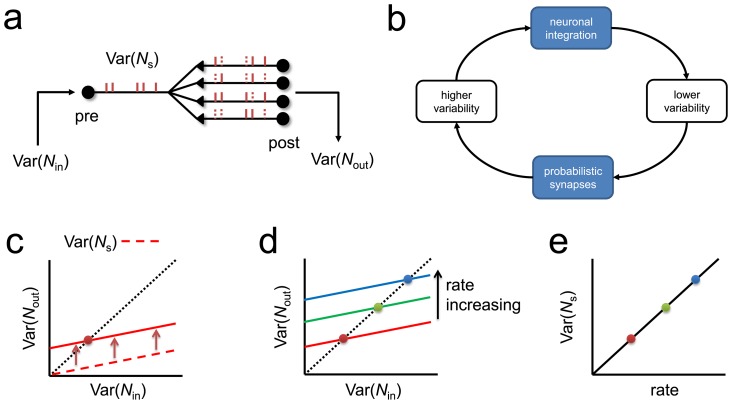
The mechanism for Poisson-like variability in a network with probabilistic synapses. (**a**) Scheme of the transformation between input variance 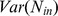 in the spike counts of the presynaptic spike trains and output variance 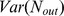 of the post-synaptic currents in an open loop network with probabilistic synapses. (**b**) Precise balancing of two competing forces in a closed-loop network: the integration step tends to lower spiking variance, while the probabilistic synaptic step increases spiking variability. (**c**) Output variance (solid red line) and the variance of the spike train, 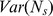 (dashed) increase linearly as a function of input variance for fixed input firing rates. Solid line is vertically shifted respect to the dashed line due to the increase of variance by probabilistic synapses, which is uniform for all input variances. The equilibrium point of the network (red point) corresponds to the state where the input and output variances match. (**d**) The equilibrium point moves linearly with firing rate because the vertical shift induced by probabilistic synapses increases linearly with rate. (**e**) Spike count variance increases linearly with rate, leading to Fano factor constancy.

To understand how Poisson-like firing arises from networks with probabilistic synapses, we used a simplified network model where transmission probability is time-independent (Fig. 5a; see Methods). A neuron in the network (pre) receives a barrage of spikes per presynaptic neuron with spike count 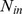 and variance 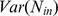 and generates an output spike train whose spike count 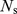 has variance 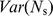. This spike train in turn evokes post-synaptic currents (PSCs) with variance 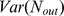 on postsynaptic cells (post). The evoked PSCs are replicas of the same presynaptic spike train that has been diluted by a fraction *p*, corresponding to the probability of synaptic transmission.

There are two competing forces that affect the variability of the spike trains and series of PSCs (Fig. 5b). The first one is the integration step of the neuron, which tends to lower the input variance. And the second one is the probabilistic synaptic step, which increases the variance. These two forces have to cancel out precisely when the network reaches equilibrium, because at equilibrium 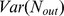 should equal 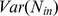. More precisely, it can be shown (see Methods) that 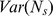 is proportional to the input variance 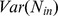 at fixed input firing rate (dashed red line, Fig. 5c) and that the effect of probabilistic synapses is shifting this line upwards regardless of the value of the input variance (solid red line). The point at which the input and output variances are the same (red dot) corresponds to the equilibrium state of the network. The crucial question is to determine how this equilibrium point depends on the rate of the network. If the firing rate of the network increases, the crossing point moves at higher values linearly with the population rate (Fig. 5d). Because the spike count variance is proportional to the output variance at equilibrium (see dashed line in Fig. 5c), the spike count variance increases linearly with population rate (Fig. 5e). Therefore, the ratio between variance and mean in the spike count is constant in this network, leading to Fano factor constancy. The same Poisson-like generation mechanism takes place in more biophysically realistic networks (Fig. 2), and holds exactly for networks of spiking neurons with probabilistic synapses with constant transmission probability (Fig. 3e; see Methods).

This mathematical exercise (see details in Methods) shows that the presence of both excitation and inhibition is not strictly necessary for Poisson-like variability, since it is possible to obtain high sustained variability in large networks with pure excitation and sufficiently weak synapses. However, although balancing strong excitation with inhibition is not required *per se* for Poisson-like variability at moderately high rates, the presence of both excitation and inhibition is required to avoid runaway excitation in networks with more realistically strong excitatory synapses [9], [37] (see Fig. 2).

Finally, other types of noise, such as random synaptic delays [1] with arbitrary distributions or ion-channel noise that jitters randomly the timing of the evoked action potentials [38], [39] do not lead to Poisson-like variability (Fig. 3d; see also Methods). Although random synaptic delays, a broad static distribution of synaptic delays [40], or spike jittering can improve the stability of Poisson-like variability, these types of noise are not sufficiently amplified by recurrent neuronal networks at high rates, and therefore they constitute a negligible source of noise.

### Membrane potential and synaptic conductance fluctuations

**Figure 6:**
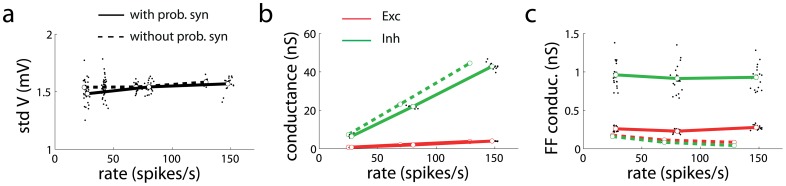
Theoretical predictions: Fano factor constancy of synaptic conductances. (**a**) The standard deviation of the membrane potential is approximately constant as a function of firing rate for networks with (full line) and without (dashed) probabilistic synapses. (**b**) The mean excitatory (red) and inhibitory (green) conductances increase linearly with firing rate. (**c**) The Fano factor of the synaptic conductances (FF, variance to mean ratio) for a network with probabilistic synapses is constant as a function of the firing rate (full lines), indicating that the variance of the conductance is proportional to the mean conductance. The FF of the synaptic conductances for a network without probabilistic synapses is lower than in the previous case and strongly decreases with firing rate (dashed lines). For all panels, open circles correspond to mean values and black dots correspond to sampled neurons. Error bars represent s.e.m.

Because probabilistic synapses introduce multiplicative noise at the synaptic level, the membrane potential and synaptic conductances of neurons must show some characteristic statistical properties. We studied these statistical properties in recurrent networks of conductance-based spiking neurons in the high-conductance regime [11], [41]. We found that the standard deviation of the neuron membrane potential is approximately constant as a function of firing rate (Fig. 6a) for both networks with (full line) and without (dashed) probabilistic synapses, consistent with experimental observations [5], [30]. The fact that the constancy of the standard deviation of the membrane potential naturally arises in neuronal networks can be used as a justification of Gaussian rectification models of single cell spiking variability, where the standard deviation is assumed to be constant with firing rate [5]. Both excitatory and inhibitory synaptic conductances increased linearly with rate (Fig. 6b). Interestingly, the Fano factor (FF, variance to mean ratio) of the conductances was approximately constant as a function of firing rate for networks with probabilistic synapses, but it was much smaller and decreasing rapidly with firing rate for networks without probabilistic synapses (Fig. 6c). These results show that the size constancy of the membrane potential fluctuations arises as a result of the shunting effect of mean conductances on the conductance fluctuations [11], [41], [42], while the FF constancy of the synaptic conductances is a natural consequence of the multiplicative noise introduced by probabilistic synapses. The FF constancy of synaptic conductances is an experimentally testable prediction of our theory.

## Discussion

We have shown that spiking networks endowed with probabilistic synapses lead to Poisson-like variability for several orders of magnitude in firing rate, in line with extensive experimental observations in sensory areas [2], [3], [4], [6], [7]. Poisson-like spiking variability naturally arises from the multiplicative nature of synaptic noise and its amplification through strong recurrent connections and does not require fine-tuning of the network parameters. The multiplicative noise implies that that the size of membrane potential fluctuations is relatively constant with firing rate while the size of synaptic conductance fluctuations grows in proportion to their means.

Other sources of variability could also contribute to evoked cortical spiking variability. Experimentally uncontrolled external variables might artificially introduce spiking variability that is not a property of the system *per se*. However, even when eye movements are controlled [6] or paralyzed [43], or when the statistical properties of the stimulus are fixed [44], cortical neurons still respond with high Poisson-like variability at all registered neuron firing rates. Photon noise and intrinsic receptors' noise can partly explain cortical spiking variability [45], but at high stimulus intensities this variability is unlikely to represent a major contribution. Internal variables such as attention and arousal might also be at place, but even when they are controlled experimentally, spiking responses are still highly variable [46], [47]. Therefore, the hypothesis that variability is intrinsically generated by neuronal networks with probabilistic synapses is favored against other less specific alternatives in view of the very little explanatory power that the presence of uncontrolled external or internal variables has on the type of spiking variability that is observed in cortex.

At the mechanistic level, a balance between strong excitatory and inhibitory inputs that sets the membrane potential below threshold has become the prevalent model for high cortical spiking variability [3], [9], [10]. Experimental evidence supports that cortical networks are in the balanced regime [48]. In evoked conditions, sensory stimulation drives individual cortical neurons to a state where the mean membrane potential increases with contrast and firing rate is high [5], [30]. As it has been shown (see Fig. 1), in this condition Fano factors are low even in the balanced regime unless input spike trains are themselves Poisson-like, raising the question as to how input Poisson-like variability is generated in the first place and whether this type of inputs is realistic. Precise cancellation of the input currents can potentially clamp the membrane potential to a value below threshold for a broad range of firing rates, but this exquisite cancellation requires fine-tuning of the network parameters for very large networks [12]. It has also been suggested that to produce *in vivo* high spiking variability, presynaptic spikes need to be synchronous [49], but it was unknown how large input variability caused by synchrony can be generated in recurrent networks. Previous models have also explored the role of synaptic noise in neuronal computations [50], [51], [52], [53], in up-down state transitions [54] and in the spiking variability of single cells or pairs of cells [50], [55], [56], [57], but the role of probabilistic synapses on Poisson-like variability in large recurrent networks or over a broad continuum of firing rates was not studied. As we have shown here, probabilistic synapses generate multiplicative noise that is amplified by recurrent connections without fine-tuning of the network parameters. This mechanism underlies a sufficient requisite for large multiplicative input fluctuations that guarantees Poisson-like spiking for several orders of magnitude in firing rate.

Probabilistic synapses have also the potential to explain at the mechanistic level the origin of high spiking variability in a much broader context than the one that we have considered here. High activity states during delayed persistent activity in working memory tasks are characterized by high spiking variability [16], [17]. Although bistable attractor networks have been shown to display high variability at both spontaneous and moderately high rate persistent activity states [12], [13], [14], [15], the contribution of probabilistic synapses to spiking variability in these networks has not been studied. We have shown that probabilistic synapses stabilize Poisson-like firing for a broad continuum of firing rates in single-attractor networks because this type of noise introduces multiplicative noise. Clearly, probabilistic synapses have also potential to account by itself for the high variability observed during persistent activity in working memory tasks. Therefore in future studies it will be important to elucidate the role of probabilistic synapses on spiking variability and stability of working memory states in bistable attractor networks.

It has been recently shown that stimulus onset reduces the average Fano factor across a broad variety of cortical areas and conditions [58], a reduction that is specific to the transition from spontaneous to evoked activity. This reduced variability has been hypothesized to arise because of the redirection of the system to a particular state configuration during stimulation [58]. It is important to realize however that despite the reduction of variability relative to spontaneous activity, the responses in evoked conditions are still highly variable and the Fano factor is approximately constant with neuron's firing rate, as it has been shown by many previous studies [2], [3], [4], [6], [7]. Simulated neuronal networks based on balanced inputs with weak multi-attractor states can account for the finding that variability is reduced at stimulus onset [12], [33], [59], [60], but they leave unanswered why the Fano factor remains approximately constant in a broad range of firing rates in evoked conditions. In the general condition as in the specific networks studied in those works, increasing the input will eventually guide neurons to the supra-threshold regime, where firing is due to quasi-deterministic membrane potential threshold crossings and Fano factors decrease with increasing firing rate (see Fig. 1b). As we have demonstrated, balanced neuronal networks with probabilistic synapses can generate Fano factor constancy for a wide range of firing rates in evoked conditions even in the supra-threshold regime because synaptic noise is multiplicatively scaled up with firing rate.

Finally, injecting noise in the brain with probabilistic synapses might seem harmful at a first glance. Therefore it can appear that we have presented a “solution” to the Poisson-like variability problem, but we have “created” a new one: boosting neuronal variability. However, noisy systems can have an advantage against deterministic systems in detecting sub-threshold stimuli [61], learning more quickly [62], and displaying larger memory capacity [63]. Injecting noise through probabilistic synapses is particularly relevant in view of the new computational capabilities that neuronal networks with Poisson-like firing acquire, allowing neuronal codes to be in the appropriate format to perform optimal cue combination [64] and sampling cortical states over the whole dynamical range [65]. Therefore, synaptic noise is not only a robust and sufficient mechanism for the type of variability found in cortex, but it can also provide cortical circuits with computational tools to perform probabilistic inference under noisy and ambiguous conditions.

## Methods

### Spiking network with probabilistic synapses

We consider a network of leaky integrate-and-fire (LIF) neurons with 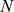 cells, 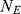 of which are excitatory and 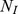 are inhibitory [10], [66], [67], [68]. The membrane potential of neuron *i* below the spiking threshold obeys
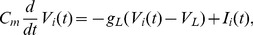
(1)where 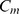 is the membrane capacitance, 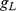 is the passive leak conductance and 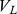 is the resting state potential. The membrane time constant of the neuron is defined as 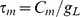.The neuron emits a spike when the membrane potential reaches the threshold 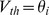, after which the potential is reset to 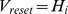. The total synaptic current 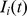 delivered to the neuron is
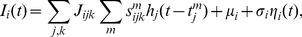
(2)where the first term corresponds to the currents generated by other cells in the network, while the two last ones correspond to the current generated by external sources to the network. 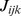 is the connectivity strength of contact *k* between the presynaptic neuron *j* and the postsynaptic neuron *i*. We typically consider 2–6 contacts per pair of connected neurons. Auto-synapses are not included in the network, i.e. 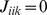 for all *i*. The sum over *m* corresponds to the spikes times of each presynaptic cell *j*, denoted 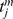, 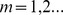. Each spike from neuron *j* can potentially generates a stereotyped current after a delay 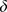 on the postsynaptic cell proportional to the synaptic kernel 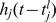, such that
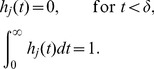
(3)With this choice, the total charge injected in the neuron due to a presynaptic spike at time 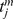 is determined by 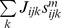, where 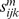 is the synaptic variable that specifies the amount of neurotransmitter released at contact *k* between postsynaptic neuron *i* and presynaptic neuron *j* at the time of the presynaptic spike 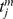. The dynamics of the synaptic variables are described by the end of this section. As synaptic kernel, we choose
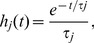
(4)where 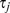 is the synaptic decay time constant of the postsynaptic current, which can be excitatory with time constant 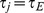 or inhibitory with time constant 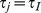. The external current consists of a deterministic component (mean drive) 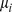, and a white noise process 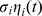 with variance 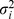. Here 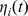 is a white noise process with zero mean and unit variance independent across neurons, i.e. 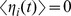 and 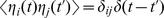, where 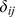 is the Kronecker's delta, and 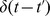 is the Dirac's delta function.

We endowed synapses with a probabilistic transmission model where the synapses evoke successfully postsynaptic currents with a fixed probability upon presynaptic spike arrival if a vesicle is ready to be released, and the replenishment of the vesicle is stochastic with an exponential distribution over time [50]. This model is based on deterministic models of short-term-depression (STD) *in vitro* [26], [27]. We further modified the deterministic models of STD for *in vitro* slices to incorporate the lack of depression observed at high rates *in vivo* [23], [25], [36] and in some neuronal population *in vitro* [69] as follows: upon vesicle release, a new vesicle is immediately ready to be released with the same probability, but with a lower neurotransmitter load. This model creates effectively a lower bound in synaptic efficacy, allowing for non-saturating responses at high firing rates. Other biophysical implementations of non-saturation and stochastic release at high rates (∼100–200 Hz) are also possible (such as, simply, very fast vesicle replenishment times), but the results of our work do not depend on the particularities of this implementation. Neuronal networks with STD models without the experimentally motivated non-saturating synapses cannot display firing above ∼50 Hz due to synaptic exhaustion with standard *in vitro* parameters, a firing rate condition in which Poisson-like variability is commonly observed in sensory areas [2], [4], [8]. Full details of the model are given next.

We first specify the dynamics of the synaptic variable 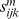, defined as the amount of neurotransmitter released at each synaptic contact 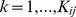 between the postsynaptic neuron *i* and the postsynaptic neuron *j* at the arrival of the *m*-th spike from neuron *j*, 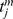. Associated to this variable, there is a neurotransmitter availability variable 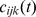 that specifies how much neurotransmitter is ready for release at any time 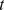.

The stochastic model of synaptic transmission at each synaptic contact is as follows and independent across contacts: (1) upon arrival of a spike at time 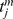 a vesicle from a readily releasable pool fuses the membrane and releases its content 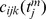 with probability 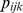. If neurotransmitter is released, the synaptic variable equals the amount of neurotransmitter that is released by the vesicle, 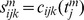, and 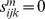 otherwise. (2) Immediately after release, a vesicle from a readily releasable pool with low neurotransmitter load becomes available. It has an amount of neurotransmitter 
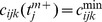
, 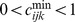. (3) The time it takes this vesicle to be replaced by a vesicle with high neurotransmitter load, 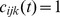, is a random variable following an exponential distribution with mean 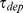.

With the above choice of the maximum value 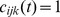, the synaptic strength at each contact is quantified by 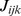. The dynamics of probabilistic synapses has been simulated as follows: for each synaptic contact that has been partially depleted to the value 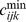, a random exponentially distributed time was generated as 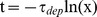 where 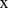 is uniformly distributed in the interval [0,1]. Once this time has elapsed, the synaptic contact was replenished to its maximum value 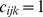. When 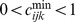 synaptic transmission is permitted immediately after a successful transmission. However, the neurotransmitter that can be immediately released is smaller than the maximum allowed value. The choice 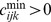 also ensures that the currents generated by the network do not saturate below 20–50 Hz.

### Network of non-leaky integrate-and-fire neurons with probabilistic synapses

We start by describing a recurrent network with non-leaky integrate-and-fire neurons (nLIF) and probabilistic synapses. In an nLIF neuron the leak term (see eq. (1)) has been dropped and the voltage obeys
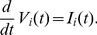
(5)For simplicity in the expression we have taken the membrane capacitance 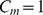. We also normalize the spiking threshold 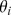 such that the reset membrane potential 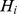 is defined to be at zero. The nLIF neuron is an excellent approximation for a LIF neuron when inputs are strong and firing rate is high, precisely the situation where Poisson-like firing breaks down in LIF networks. Therefore, showing that networks of nLIF neurons with probabilistic synapses give rise to Poisson-like firing will mean that the same property holds for LIF networks with probabilistic synapses. In the main text we show that the qualitative results derived for networks of nLIF neurons also apply to networks of LIF neurons.

The total synaptic current 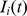 delivered to the neuron is
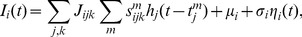
(6)identical to eq. (2) but where we use instead a simplified model of probabilistic synapse. Specifically, each synaptic variable 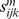 in eq. (6) becomes one with probability *p* upon spike arrival, and otherwise it is zero, independently across contacts and time. Therefore, in this model the temporal dynamics of synapses is neglected, but the probabilistic nature of synaptic transmission is preserved. The theory that is presented below is valid for any arbitrary synaptic kernel with the properties described in eq. (3). It is worth emphasizing that neglecting the temporal dynamics of synapses modifies the precise values of the steady-state neurons' firing rates and their Fano factors, but the qualitative effects about Poisson-like variability naturally extend to the more realistic case with synaptic dynamics. In the next section we compute exactly the mean activity and covariance of the spike counts across neurons in the network, required to show that a nLIF neuronal network with probabilistic synapses display exactly Poisson-like variability.

First, we rewrite eqs. (5) and (6) in a more convenient way that will highlight the effect of membrane potential resetting. Since the effect of a spike emitted by neuron *i* is to decrease its membrane potential instantaneously from threshold to the reset values, eq. (5) can be expressed as
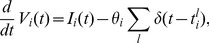
(7)where 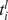 denotes the spike times of neuron *i*. Eqs. (5) and (6) can be rewritten in matrix notation as
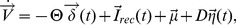
(8)where 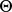 and 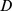 are diagonal matrices with entries 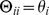 and 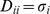. In the expression, 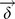, 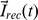 (recurrent part of the total current) and 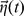 are vectors with *i*-th components 
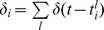
,
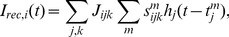
(9)and 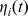, 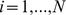, respectively. The advantage of using eq. (8) instead of eq. (5) is that the non-linear resetting mechanism of the cells is transformed into a term indistinguishable from self-inhibition or negative-feedback.

Now we move to compute the mean spike counts over the neurons in the network. In the following we assume that there is a single attractor state of the system. We start by taking expected values (over all realizations of the white noise processes and initial conditions of the network leading to the same set of active neurons) in the two sides of eq. (8) to obtain
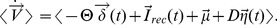
(10)Since the average membrane potential does not change in the stationary regime if the firing rates of the neurons are positive, the l.h.s. of the equation is zero. Noting that 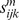 are random variables independent of spike times and both across contacts and synapses, and using that 
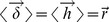
, where 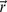 is the population firing rate vector, if the rates are non-negative we find that eq. (10) is equivalent to the constraint over the population firing rate vector
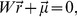
(11)where the effective connectivity matrix 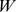 has diagonal entries 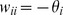 and off-diagonal entries 
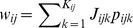
. The matrix 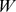 explicitly shows the self-inhibitory effect of the reset mechanism. If 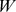 is invertible, eq. (11) can be readily solved to give an expression for the population firing rate
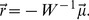
(12)
Eq. (11), written more generally to include cases where the firing rates can be zero, becomes
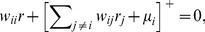
(13)where 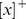 is the linear rectified function (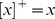 if 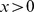, and 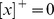 otherwise). Although we have assumed the presence of a single attractor, this equation allows for multiple solutions in general. In those cases, multi-stability develops in the network, and each state obeys an equation like eq. (11) where the connectivity matrix 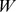 becomes the original one but where the columns and rows corresponding to the inactive neurons have been removed. The firing properties described below hold for each state but in addition stochastic transitions between the states are possible.

Eq. (11) expresses the required balance between excitatory (*E*), inhibitory (*I*) and external inputs in the stationary regime. We can rewrite this equation for the simple but illustrative case where all neurons in population 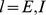 connects with all other neurons but itself in the population 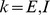 with strength 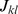, the mean input currents to the *E* and *I* populations are 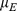 and 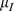, respectively, spiking threshold for all neurons is 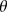, and there is a single contact per neuron pair with the same transmission probability *p* for all synapses. In this case eq. (11) it is equivalent to the set of linear equations
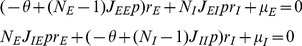
(14)Where 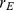 and 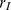 are the firing rates of *E* and *I* neurons. The first (second) equation describes the mean input currents to an *E* (*I*) neuron, and it shows that the mean excitatory, inhibitory and external mean currents and the effective depolarizing current proportional to 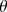 cancel precisely in such a way that the sum of them is precisely zero. Therefore, in the stationary regime the network settles down in a set of firing rates that satisfies precisely this balance equation [9]. Note that if the connectivity strengths are large (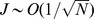), the cancelation mainly occurs between a large excitatory mean drive by a large inhibitory mean drive. Fig. 2e shows how the mean currents are dynamically cancelled, leading to a state of low firing rates.

As a next step, it is useful to show that the average activity over realizations of the white noise process equals its temporal average. We first integrate eq. (8) from time zero to a long time 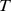 as
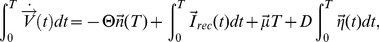
(15)where 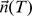 is the spike count population vector in the time window 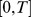 with components 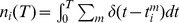, 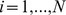. The integrated current has components
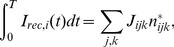
(16)where 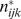 is the number of successful synaptic transmissions at contact *k* from neuron *j* to *i*. This number is a random number that depends on the number of actual spikes from neuron *j*, 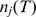, as
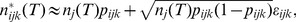
(17)where 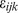 is a normally distributed variable independent across synaptic contacts. The first term in the equation corresponds to the average number of successful spike transmissions at the synaptic contact *k* from neuron *j* to *i*, while the second term correspond to the fluctuations of the number of successful transmissions around the mean, which becomes a Gaussian variable for long 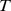. Note that the mean and variances of the Gaussian are proportional to the mean and variances of a Bernoulli process with success probability 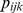. Strict equality of eq. (17) holds for 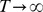.

Now it is easy to extract useful information from eq. (15). For long 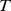 the terms proportional to 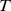 and to the spike counts 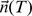
dominate. Therefore, for 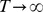 and positive spike counts eq. (15) reduces to
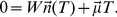
(18)This equation is identical to eq. (11) if the expression is divided by 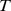, hence showing the equivalence between realization and temporal averages.

Finally, we compute the covariance matrix of the spike counts across pairs of neurons using the previous equations. We start by splitting the recurrent term 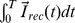 in eq. (15) into deterministic and fluctuation terms and lumping together the normally distributed random variables across contacts at each synapse. It can be shown that eq. (15) can be rewritten in a more convenient way as
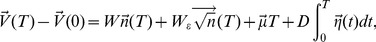
(19)where 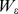 is a random matrix with entries
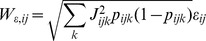
(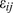 are i.i.d. normally distributed variables), and 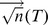 is a vector whose components are the squared root of the spike counts up to time 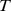, 
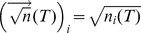
, 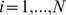. Taking expectations in both sides of eq. (19) we find
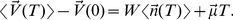
(20)Subtracting eq. (20) from eq. (19), and defining the membrane potential and spike count fluctuations as 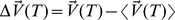
and 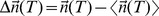 respectively, we obtain
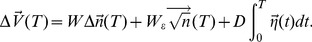
(21)This equation is the basis to obtain the covariance matrix of the spike counts. Solving for 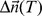 in eq. (21), we find after some laborious algebra that the covariance of the spike count can be written up to order 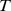 as
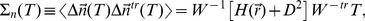
(22)where 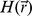 is a rate-dependent diagonal matrix with entries
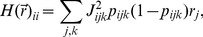
(23)and “*tr*” stands for matrix transpose.

We note that in eq. (22) the noise introduced by probabilistic synaptic transmission is multiplicative with the rate (see eq. (23)), and that it enters as a diagonal matrix that is further amplified and transformed by the recurrent connections. These results fully and exactly describe the first and second-order firing statistical properties of nLIF recurrent networks, opening in turn the door to study correlations in spiking recurrent networks with probabilistic synapses.

The probabilistic synaptic model that we have considered so far does not have variability in the amplitude of the synaptic strength. It is possible to include this source of variability in the present formalism by replacing 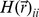 in eq. (23) by
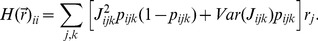
(24)where 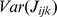 is the variance of 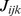 across successful synaptic transmissions at each contact, and by replacing 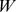 in eq. (22) by a matrix with diagonal entries 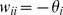 and off-diagonal entries 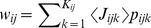.

The expression for the firing rate and covariance of the spike counts, eqs. (11) and (22), have been derived for the case where delays are fixed and there is not jittering during the generation of the spikes. However, it is possible to show that the same expressions hold when the delays and jitters are random with finite first and second order moments. This shows that noise introduced by random synaptic delays and spike generation jittering constitute a negligible source of noise in nLIF networks.

From the equation of the mean firing rates, eq. (12), and the expression for the covariance matrix of the spike counts, eq. (22), it follows that that the Fano is constant for all firing rates. If the input drive is scaled by a factor 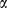, 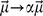, then according to eq. (12) the firing rates are scaled up by the same factor, 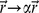. Similarly, if the noise from external sources is small, then the covariance matrix of the spike counts is approximately scaled up by the same factor, 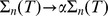 because the noise introduced by probabilistic synapses is multiplicative. Since the Fano factor is defined as 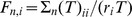, scaling up the input drive can modulate the firing rate of individual neurons by several orders of magnitude while their Fano factor remains constant. This finally shows that Poisson-like variability arises in spiking networks with probabilistic synapses by virtue of the multiplicative nature of synaptic noise.

### Mechanism for Poisson-like variability

In this section we provide details for Fig. 3 in the main text. Let us assume that the output spike train of the presynaptic neuron, described by the spike count 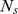, has a mean count 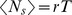 and variance 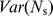, where 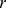 is the firing rate of the neuron and 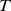 is the length of the time window. When this spike train passes through a probabilistic synapse with a probability 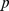 of successful transmission, a sequence of PSCs is generated with mean count 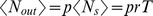 and variance 
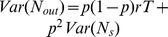
. Note that the output firing rate has been diluted by a fraction 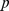, and that the variance contains two terms arising from a doubly stochastic process: the first term comes from the extra variability introduced by the probabilistic synaptic transmission, while the second term is a diluted version of the presynaptic spike train variability. It is crucial to realize that the first term is proportional to the firing rate in the network, while the second term is rate-independent. To close the loop, we need to specify the way that input mean count and variability are transformed into the mean spike count and variability of 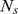. Assuming an homogeneous network of 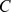 neurons with connectivity strength 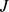, then a LIF neuron with threshold 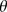 generates a spike train with mean count 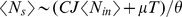 and variance 
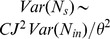
, where 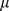 is the mean external input drive to the neurons in the network. To derive the expression for the mean and variance, we have assumed that spike trains across neurons are approximately independent and that the firing rate is high. Finally, using the relationship between 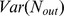 and both rate and 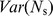, and the fact that input and output variances should be equal in a self-consistent recurrent network, we arrive to the expression 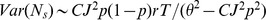. Note that this expression predicts that the spiking variability of the neurons is Poisson-like because it is proportional to the firing rate 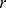 in the network. Note also that the Fano factor increases with the connectivity strength, and with the number of connections per neuron at fixed connectivity strength.

### Network and stimulus parameters

Finally, we provide details about the parameters used in each figure. The parameters for Fig. 1 are as follows. A single neuron obeying eqs. (1)–(2) was simulated. The membrane capacitance, leak conductance and leak potential were 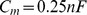, 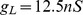 and 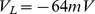 respectively. With those choices, the membrane time constant was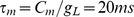. The spiking threshold and reset membrane potentials were set at 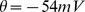 and 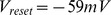 respectively [11]. For the case with constant input noise (dashed lines), the standard deviation of the noise was set at 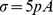. For the case of Poisson-like inputs (solid lines), the variance of the noise grew proportionally with the mean input drive 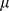 as 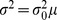, where we chose 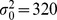.

The parameters of the network with probabilistic synapses described in Fig. 2 (solid lines) were as follows. A total of 2000 neurons were simulated, 80% of which were excitatory and 20% were inhibitory. The connectivity was all to all. The membrane capacitance, leak conductance, leak potential, and resting potential were as in Fig. 1, while 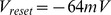. The connectivity strength 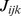 of contact *k* between the presynaptic neuron *j* in population *E* and the postsynaptic neuron *i* in population *E* took the values 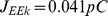, between *E* and *I* neurons 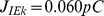, between *I* and *E* neurons 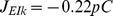, and between *I* and *I* neurons 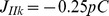. There were a fixed number of 4 contacts between all pairs of neurons. With these values, a successfully transmitted *E* presynaptic excitatory spike generates an EPSP of 0.66 mV on *E* postsynaptic neurons [34], [35]. The synaptic decay time constant of excitatory and inhibitory PSC (see eq.(4)) was 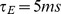 and 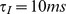 respectively. The probability of release was 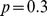 [22], the recovery time constant of vesicles took the value 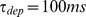 [26] and the minimum vesicle neurotransmitter fractional load was set at 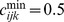 [25], [69], identical for all contacts. The mean input current 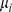 in eq. (2) was the same for all the neurons, and the input variance 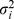 was taken to be zero. Simulations were run for 200 s with a one-step Euler method with time step 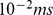. Fano factors were computed using time windows of 2 s. None of the results presented depend critically on the values of the parameters chosen.

For the network without probabilistic synapses in Fig. 2 (dashed lines), the parameters were as above except for the following. Neurons were driven by noise with constant standard deviation of the noise 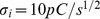. We set 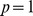 and 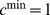, and therefore probabilistic synapses and STD temporal dynamics were absent. To produce comparable rates to those in the network with probabilistic synapses we compensated the larger 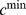 by reducing by half all synaptic strengths.

In Fig. 3a the parameters were as in Fig. 2 for the network without probabilistic synapses. The network connectivity was random and sparse, with every neuron in the network receiving connections from a small fraction, 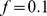, of pre-synaptic excitatory and inhibitory neurons randomly chosen. The strength of the connections was as before. In Fig. 3b, the parameters were as in Fig. 2 for the network without probabilistic synapses, with the exception that the reset potential was set at a higher value, 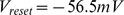. In Fig. 3c the parameters were as in Fig. 2 for the network with probabilistic synapses, but with 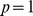 and 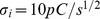. In Fig. 3d, parameters are as in Fig. 3c with the addition of a random delay independently for each spike (spike jitter) and uniformly distributed between 0 and 10 ms. In Fig. 3e the parameters were again as in Fig. 2 for the network with probabilistic synapses, except that 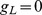, 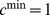 and the synaptic weights were divided by half.

In Fig. 4 parameters were identical as in Fig. 2 when the synaptic scaling factor *g* is 1. For the cases of scaling factor different from one, all connectivity strengths of the network in Fig. 2 were multiplied by *g*, keeping fixed all other parameters.

For the conductance-based network with probabilistic synapses in Fig. 6, parameters were as in Fig. 1, with 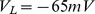 and 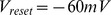. Synaptic current were modeled as 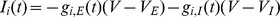, where 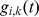 (k = *E*,*I*) is the synaptic conductance and the reversal potentials are 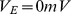 and 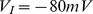. The synaptic conductances follow an equation identical to eq. (2) with 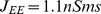, 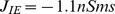, 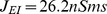 and 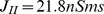. Probabilistic synapses without STD were studied with 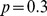. For the network without probabilistic synapses (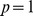) all synaptic strengths were reduced three-fold to keep firing rates close to those from the network with probabilistic synapses. In addition, external constant noise was added to each neuron with standard deviation 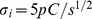. In both networks, there were 100 neurons with a single contact between all pairs of neurons, of which 80 were excitatory and 20 were inhibitory. The synaptic decay time constant of excitatory and inhibitory PSC (see eq. (4)) was 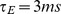 and 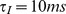 respectively. External current-based inputs were excitatory and identical to all neurons.
